# A Migrant Health Package for Providing Psychiatric Care to People on the Move in Mexico and Central America

**DOI:** 10.1192/bjo.2026.11587

**Published:** 2026-06-30

**Authors:** Lindsay Solera-Deuchar, Carolina Echeverri, Marcos Moyano, Cristina Carreño, Laura Moreno

**Affiliations:** 1 East London NHS Foundation Trust, London, United Kingdom; 2 Médecins Sans Frontières, Geneva, Switzerland; 3 Médecins Sans Frontières, Barcelona, Spain; 4 Servicio Madrileño de Salud, Madrid, Spain

## Abstract

**Aims:**

There are significant challenges to providing psychiatric care to people on the migratory route in Central America, including the low probability of face-to-face follow-up. However, there is a clear need for psychiatric care, for both pre-existing psychiatric conditions, and new onset conditions, some related to stressors experienced on the migratory route.

There is no literature providing clear guidance as to how to provide psychiatric care to people on the move. Therefore, this quality improvement project had the following aims:

1. To provide clinicians with greater clarity as to whether to offer psychiatric medication to people on the move

2. To improve continuity of care for people on the move taking psychiatric medication

**Methods:**

Through a consultation process, four Médecins Sans Frontières (MSF) psychiatrists created five flowcharts to aid clinicians in assessing the benefits and risks of providing psychiatric treatment to a person on the move, in collaboration with the person. The flowcharts were piloted by clinicians, evaluated, and improved in a second cycle of change.

In consultation with mental health teams and patients from MSF’s projects across the region, a migrant health package was created, including psychoeducation leaflets, detailed medication labels, a regional map of clinics (MSF and non-MSF) providing psychiatric care on the migratory route, and a referral form. The package was implemented across four of MSF’s project sites.

**Results:**

A survey revealed that the flowcharts improved clinician confidence in deciding whether it was appropriate to prescribe psychiatric medication to people on the move.

In 2023, 78 patients were provided with the migrant health package. There were challenges in assessing whether the intervention increased continuity of care, as in many cases contact with the person was lost, and currently there is no regional patient database across MSF projects. However, of the 78 patients, 21% (16) were confirmed to successfully receive follow up at a clinic further along their route.

**Conclusion:**

The project provided clarity for clinicians as to when it is appropriate to offer psychiatric medication to a person on the move. Further work is needed to evaluate whether the migrant health package improves continuity of care for people on the move receiving psychiatric care from MSF in Mexico and Central America. However, at least 21% of patients provided with the package were able to access face-to-face follow up following initial contact.

